# Non-employment over the working life: Implications for cognitive function and decline in later life

**DOI:** 10.1016/j.puhip.2024.100563

**Published:** 2024-12-23

**Authors:** A.J. Sizer, A. Sacker, R.E. Lacey, M. Richards

**Affiliations:** a Department of Epidemiology and Public Health, University College London, London, UK; b Research Department of Epidemiology and Public Health, University College London, London, UK; c MRC Unit for Lifelong Health and Ageing at UCL, University College London, London, UK

**Keywords:** Ageing, Cognition, Birth cohort study, Life course epidemiology

## Abstract

**Objectives:**

Disuse theory predicts that cognitive function is vulnerable to transitions that remove factors that support cognitive skills. We sought to investigate whether non-employment over the working life was associated with cognitive function and decline in later life (≥60 years old), and possible gender differences in the association.

**Study design:**

Longitudinal study.

**Method:**

We used data from the MRC National Survey of Health and Development (NSHD). Cognitive function was measured by verbal memory and processing speed. Linear regression was used to test associations between non-employment duration and cognitive function at age 60–64, and conditional change models were used to examine associations between non-employment and cognitive decline from age 60–64 to 69. Gender specific models were adjusted for childhood factors and educational attainment, adult occupational features, and adult health and lifestyle indicators. Missing data was accounted for using multiple imputation by chained equations.

**Results:**

In fully adjusted models >15 years non-employment was associated with lower cognitive function at age 60–64 in men (verbal memory: −0.72, 95%CI −1.18, −0.26; processing speed: −0.61, 95%CI -1.00, −0.28), but not women. Fully adjusted models also indicated that long-term and intermediate lengths of non-employment were associated with faster decline in verbal memory (−0.38, 95%CI -0.75, −0.02) and processing speed (−0.28, 95%CI -0.52, −0.03) in men. There was no association between non-employment and cognitive decline among women.

**Conclusion:**

Long-term non-employment in men, but not women, is associated with accelerated cognitive ageing.

## 1 Introduction

Cognitive abilities are integral to health and well-being throughout life, and their maintenance into old age is an important determinant of quality of life and living independently. Cognitive skills develop throughout life, and while cognitive decline can be a normal part of ageing, rates of decline show considerable variability between individuals [1], and are influenced by a range of factors that operate across the life course [2] including social class [3], education [4], health [5], and lifestyle [6].

Disuse theory [7,8] suggests that cognitive function is vulnerable following transitions that remove factors that support cognitive skills. In addition to physical activity, social interaction and daily goal structure, employment provides a variable source of cognitive stimulation, all of which can influence cognitive function. Therefore, removal of work-based cognitive exposures during periods out of employment may decrease opportunities to participate in activities influencing cognitive function and decline in later life. Consistent with this, the Kohn-Schooler research programme [9,10] demonstrated that work provides a variable source of intellectual demands (measured by job complexity) and that these demands are associated with cognitive functioning twenty years later, even after controlling for prior cognitive ability [11]. These findings have been replicated in different study populations, using different measures of occupational complexity and different cognitive measures [[12], [13], [14], [15], [16]].

Despite the large body of research that has examined job complexity and cognition in mid and later life, relatively little has examined the association of economic inactivity over the working life (hereafter called non-employment) with cognitive functioning in later life. Risk of cognitive impairment was higher in individuals experiencing non-employment spells lasting six months or more [17], and the duration of economic inactivity among individuals aged 50 and above was negatively associated with verbal recall [18]. However, these studies did not examine if these associations differed between men and women. To address this gap, using the MRC National Survey of Health and Development (NSHD) this paper aims to.

1. Examine the association between non-employment duration and cognitive function and decline in later life. We hypothesised that non-employment represented disuse through the removal of employment-related factors that influence cognition, therefore higher non-employment durations would be associated with lower cognitive function and faster cognitive decline in later life.
2. Investigate whether associations differed by gender. We hypothesised that associations between non-employment and cognitive function and decline would be stronger in men than women, owing to differing gendered socioeconomic expectations and the division of work [19].

## 2 Methods

### 2.1 Study population

Participants for this longitudinal study were drawn from the NSHD, a social class stratified sample originally consisting of 5362 singleton births within marriage, in one week in 1946 in mainland UK [20]. At age 60–64, participants were shown to be representative of the population of single births to married women in the early post-World War II era, except that those not interviewed were more socially disadvantaged, had lower childhood cognitive ability, and poorer health [21,22].

The analytic sample included participants assessed at 60–64 years, with valid data on cognitive function (age 60–64 and 69), non-employment duration and the covariates that were used in the analyses. Of the 2229 NSHD participants assessed at 60–64 years, 2147 and 2179 had valid data on verbal memory and processing speed respectively, and of these 359 and 355 were missing these at age 69. Of these study members, 366 (verbal memory) and 361 (processing speed) were missing non-employment duration, and a further 523 (verbal memory) and 532 (processing speed) were missing data on the covariates.

### 2.2 Measures

#### 2.2.1 Cognitive function and decline

At ages 60–64 and 69 cognitive tests were administered by trained personnel. Verbal memory was assessed using a 15-word learning task with three recall trials (one delayed), giving a maximum possible score of 45. Processing speed was assessed by a timed letter search task. Participants searched for two target letters in a letter grid. Processing speed was the number of letters searched in 1 min [23].

To facilitate comparisons across the cognitive measures the test scores were standardised based on the relevant cognitive scores for the entire sample.

#### 2.2.2 Non-employment

Non-employment duration between leaving full-time education and age 60–64 was based on information collected from participants at ages 36, 43, 53 and 60–64. At ages 36 and 43 study members provided the start and end dates of non-employment spells lasting ≥3 months since leaving full-time education and since last contact, respectively. At ages 53 and 60–64 participants stated how much of the time they had not been in any paid work since the previous wave, categorised into: none; 1–5 months; 6–11 months; 1–4 years; 5–9 years; and continuously since age 43 (at age 53) and age 53 (at age 60–64). From these data lifetime non-employment was derived and grouped into up to 1 year; >1–5 years; >5–10 years; >10–15 years; >15 years.

#### 2.2.3 Covariates

Potential confounders were father's social class [24]; childhood cognitive ability (age 8) [24]; adolescent mental health [25]; and highest educational qualification. Father's social class was from age 11 or, where this was missing, age 15 or four. It was categorised according to the UK Registrar General's Social Class (RGSC) schema. Childhood cognitive ability was based on four tests taken at age 8. Test scores were standardised to the whole population and averaged to provide an overall cognitive ability measure. Adolescent mental health was assessed at age 13 and 15 by teachers using a questionnaire from which three mental health dimensions were identified: self-organisation; emotional problems; and conduct problems [25]. Higher scores on the emotional and conduct dimensions indicated increasing problems, whereas higher scores on the self-organisation dimension indicated fewer problems. Highest educational qualification was based on the highest education qualification achieved by age 26.

Potential mediators measured later in the life course (age 60–64) were head of household social class and occupational complexity; physical and mental health; and lifestyle. Head of household social class was based on the RGSC. For men and unmarried women this was based on their own social class, but for married women it was based on own or husband's social class, whichever was higher. Occupational complexity was proxied by the National Statistics Socio-economic Classification (NSSEC). Seven NSSEC analytic classes were used (higher managerial, administrative and professional occupations; lower managerial, administrative and professional occupations; intermediate occupations; small business employers and own account workers; lower supervisory and technical occupations; semi-routine occupations; and routine occupations). For those not working at age 60–64, NSSEC was measured by their previous known job.

Physical health variables were self-reported hypertension and heart trouble, and nurse measured systolic blood pressure (SBP), lung function (FEV_1_), and body mass index (BMI). Mental health was assessed using the 28-item General Health Questionnaire (GHQ) [26], which had four possible responses ranging from better than usual [1] to much worse than usual [4]. These were dichotomised (1, 2 recoded to 0, and 3, 4 recoded to 1), and summed giving a total score of 0–28 (higher scores represented worse mental health). Lifestyle measures were cumulative smoking intensity, estimated using pack years to age 60–64 [27]. Physical activity was estimated at age 36, 43, 53 and 60–64 by asking about spare time participation in sports and vigorous leisure activities, categorised as inactive (0), 1–4 times in the previous month (1), and ≥5 times in the previous month (2). The scores at each age were averaged and rounded to whole numbers resulting in a five-category variable (higher scores indicated higher intensity of physical activity). Social activity at age 60–64 was estimated by participation in spare time activities in the previous month (none; 1 activity/month; 2 activities/month; ≥3 activities/month [28].

### 2.3 Statistical analyses

Linear regression was used to examine the associations of interest and a series of multivariable models was developed to include those covariates that were associated with verbal memory or processing speed at age 60–64 and improved the fit of model 1^1^ (below).

Model 1: cognitive function regressed on non-employment duration.
  − Model 2: model 1 adjusted for childhood social class, childhood cognitive ability, adolescent mental health, and educational attainment.
  − Model 3: model 2 adjusted for adult social class and occupational complexity.
  − Model 4: model 2 adjusted for physical health (self-reported hypertension and BMI) and mental health.
  − Model 5: model 2 adjusted for lifestyle (smoking intensity, physical activity and social activity).
  − Final model: adjusted for the variables in the above models.

To ensure comparability, the same models were used for both cognitive function measures, and men and women.

Conditional models of change were used to examine associations between non-employment duration and cognitive decline between age 60–64 and 69. Cognitive change was derived by subtracting scores at age 60–64 from age 69 scores, which was then regressed on non-employment duration, adjusted for cognitive function at age 60–64 (Model 1).

The analytic models were developed using the same stages of adjustment as for cognitive function, and the same models were used for both measures of cognitive decline and men and women. The change scores were standardised based on the change scores for each cognitive measure for the entire sample. Positive coefficients represented a slower rate of cognitive decline, while negative coefficients represented a faster rate of decline.

All analyses were stratified a priori by gender.

### 2.4 Multiple imputation

Failing to account for missing information in longitudinal studies can lead to bias since those who are more disadvantaged or less healthy are more likely to be under-represented in complete case analyses. Missing data were accounted for using multiple imputation by chained equations (MICE) [29], were weighted and imputed separately for men and women. Forty imputed datasets were created. Descriptive statistics for the covariates before and after imputation were substantively similar for men and women (Supplementary Material Table 1).

The analyses were restricted to those with observed outcome values [30], 1788 (verbal memory) and 1824 (processing speed) individuals.

Stata v.14 [31], was used for the multiple imputation and all the analyses.

## 3 Results

### 3.1 Sample characteristics

Cognitive function at age 60–64 and 69 was lower for men than women on verbal memory and processing speed, (Table 1).

**Table 1:** Observed cognitive function scores (age 60–64 and age 69) for the sample.

Variable	Men		Women	
Cognitive function	Mean	95 % CI	Mean	95%CI
Verbal memory (age 60–64)	22.68	22.23, 23.14	24.92	24.48, 25.37
Verbal memory (age 69)	20.78	20.32, 21.24	22.84	22.40, 23.28
Processing speed (age 60–64)	259.51	253.99, 265.04	267.83	262.17, 273.50
Processing speed (age 69)	252.20	246.69, 257.71	263.86	258.25, 269.47

Short non-employment durations (≤12 months) were more common in men than women, and longer durations (>10 years) were more common in women than men (Table 2). Table 2 also shows descriptive statistics for the sample by gender. It shows that for some variables there were significant differences between men and women, including adolescent mental health, educational attainment, social class, occupational complexity, adult mental health and smoking intensity. (Table 2).

**Table 2:** Descriptive statistics for the sample, by gender.

Variable	Men	Women	Total
	Mean or %	Mean or %	Mean or %
	95%CI	95%CI	95%CI
**Non-employment**			
0–12 months	50.47	15.94	32.19
	46.23, 54.71	13.04, 18.83	29.49, 34.89
>1–5 years	23.29	24.81	24.1
	19.71, 26.88	21.40, 28.22	21.63, 26.57
>5–10 years	15.77	22.67	19.42
	12.64, 18.89	19.23, 26.12	17.08, 21.77
>10–15 years	6.84	16.20	11.80
	4.61, 9.07	13.05, 19.34	9.79, 13.80
>15 years (men) > 15–20 years (women)	3.63	20.38	12.52
	1.99, 5.26	17.08, 23.68	10.57, 14.47
>20 years (women)		8.31	8.31
		6.22, 11.03	6.22, 11.03
**Social class of origin:**			
I: Professional and managerial	5.09	3.63	4.32
	3.75, 6.44	2.61, 4.65	3.49, 5.15
II: Intermediate	16.60	17.33	16.98
	13.77, 19.43	14.56, 20.10	15.00,18.97
IIINM: Skilled non-manual	10.36	11.29	10.86
	8.36, 12.36	9.15, 13.44	9.39, 12.32
IIIM: Skilled manual	43.28	44.63	43.99
	39.10, 47.46	40.66, 48.61	41.11, 46.88
IV: Semi-skilled	16.59	17.75	17.21
	13.45, 19.74	14.74, 20.76	15.03, 19.38
V: Unskilled	8.08	5.36	6.64
	5.65, 10.50	3.45, 7.27	5.11, 8.17
**Childhood cognitive ability**	0.005	0.06	0.03
	−0.006, 0.07	−0.003, 0.12	−0.10, 0.77
**Adolescent mental health:**			
Self-organisation problems	0.98	1.40	1.20
	0.86, 1.09	1.30, 1.51	1.13, 1.28
Emotional problems	−0.22	0.02	−0.09
	−0.34, −0.11	−0.87, 0.13	−0.17–0.01
Conduct	0.20	−0.07	0.06
	0.09, 0.32	−0.19, 0.05	−0.03, 0.14
**Educational attainment:**			
None attempted	34.92	35.34	35.14
	30.90, 38.94	31.53, 39.15	32.38, 37.91
Sub-GCE or equivalent	5.61	11.85	8.92
	3.65, 7.57	9.20, 14.50	7.23, 10.60
GCE O′ Level or equivalent	14.99	26.03	20.83
	12.05, 17.92	22.61, 29.45	18.53, 23.13
GCE A′ Level or equivalent	31.00	22.35	26.42
	27.18, 34.81	19.30, 25.41	23.99, 28.86
1st degree or graduate equivalent	13.48	4.43	8.69
	10.94, 16.02	3.01, 5.84	7.27, 10.11
**Head of household social class:**			
I: Professional and managerial	12.51	9.80	11.08
	9.94, 15.08	7.58, 12.03	9.39, 12.77
II: Intermediate	38.93	47.14	43.28
	34.95, 42.92	43.27, 51.02	40.49, 46.07
IIINM: Skilled non-manual	10.37	25.78	18.53
	7.86, 12.87	22.32, 29.22	16.30, 20.75
IIIM: Skilled manual	26.00	9.05	17.03
	22.25, 29.75	6.74, 11.36	14.82, 19.23
IV: Semi-skilled	8.15	5.26	6.62
	5.81, 10.49	3.49, 7.03	5.17, 8.06
V: Unskilled	2.24	1.73	1.97
	0.90, 3.57	0.66, 2.80	1.12, 2.81
Not working	1.80	1.24	1.51
	0.64, 2.96	0.34, 2.15	0.78, 2.23
**Occupational complexity:**			
Higher managerial, administrative and professional	19.50	3.17	10.85
	16.44, 22.57	1.89, 4.44	9.21, 12.49
Lower managerial, administrative and professional	22.20	27.29	24.90
	18.92, 25.48	23.93, 30.64	22.54, 27.25
Intermediate	6.42	24.61	16.05
	4.46, 8.37	21.25, 27.98	13.98, 18.12
Small employers and own account workers	19.81	10.55	14.90
	16.52, 23.09	8.15, 12.94	12.89, 16.92
Lower supervisory and technical	9.79	3.09	6.24
	7.25, 12.33	1.69, 4.48	4.82, 7.66
Semi-routine	11.10	21.42	16.56
	8.41, 13.79	18.17, 24.67	14.41, 18.72
Routine	11.18	9.88	10.49
	8.51, 13.85	7.49, 12.27	8.71, 12.27
**Heart problems:**			
No	74.95	74.20	74.55
	71.27, 78.63	70.75, 77.66	72.03, 77.08
Yes	25.05	25.80	25.45
	21.37, 28.73	22.34, 29.25	22.92, 27.97
**Hypertension:**			
No	63.66	63.38	63.51
	59.63, 67.69	59.56, 67.21	60.73, 66.30
Yes	36.34	36.62	36.49
	32.31, 40.37	32.79, 40.44	33.70, 39.27
**Systolic blood pressure (mmHg)**	143.35	135.58	139.24
	141.77, 144.93	134.10, 137.06	138.14, 140.34
**Forced expiratory volume in 1 s (litres)**	3.05	2.12	2.56
	3.00, 3.11	2.09, 2.16	2.52, 2.60
**BMI (Kg/m**^**2**^**)**	27.92	28.45	28.2
	27.61, 28.24	27.99, 28.91	27.92, 28.49
**GHQ-28**	1.74	2.85	2.33
	1.49, 2.00	2.50, 3.20	2.11, 2.55
**Smoking intensity**	12.84	9.73	11.19
	11.30, 14.37	8.46, 11.00	10.19, 12.19
**Physical activity:**			
Inactive at all ages	23.91	28.73	26.46
	20.31, 27.52	25.14, 32.32	23.91, 29.01
Inactive/low activity (all ages)	27.56	31.31	29.55
	23.70, 31.43	27.62, 35.00	26.89, 32.21
Low-moderate activity	24.70	20.58	22.52
	21.11, 28.29	17.43, 23.73	20.15, 24.89
Moderate activity	17.14	14.70	15.85
	14.03, 20.25	11.92, 17.48	13.78, 17.92
Highly active (all ages)	6.68	4.67	5.62
	4.71, 8.66	3.02, 6.33	4.34, 6.90
**Social activity:**			
None	24.58	23.49	24.00
	20.86, 28.30	20.04, 26.94	21.46, 26.53
1 activity/month	27.33	24.16	25.65
	23.53, 31.13	20.71, 27.61	23.08, 28.22
2 activities/month	23.82	22.21	22.97
	20.22, 27.42	18.93, 25.50	20.51, 25.43
3+ activities/month	23.28	30.14	27.38
	20.82, 27.72	26.60, 33.69	24.88, 29.88

### 3.2 Non-employment duration and cognitive function at age 60-64

Cognitive scores were lower for men experiencing long non-employment durations; men non-employed for >15 years had a verbal memory score that was 0.72 s d. units lower than men non-employed for 0–12 months. Adjustment for childhood covariates (model 2), and the indicators of disuse (model 3) attenuated the association. Adjustment for health and lifestyle covariates had little effect. The unadjusted model also suggested that ≥1–5 years non-employment was associated with higher verbal memory (0.18, 95%CI: −0.01, 0.37), but this association was largely explained by childhood factors (model 2). Unadjusted differences in verbal memory for other non-employment intervals were marginal. Long non-employment durations in men were also associated with lower processing speed (0.61 s d. units, 95%CI: −1.00, −0.23), which was partially attenuated by childhood factors (model 2) and additionally by lifestyle factors (model 5). The indicators of disuse had little effect on the association, and in the final model the association remained (−0.51, 95%CI: −1.02, −0.004) (Table 3).

**Table 3:** The association between non-employment and cognitive function (standardised).

Non-employment duration *(reference category 0–*12 months *omitted)*		Model 1	Model 2	Model 3	Model 4	Model 5	Final model
		β	β	β	β	β	β
		95%CI	95%CI	95%CI	95%CI	95%CI	95%CI
**MEN**							

**Verbal memory (n = 854)**	1–5 years	0.18	0.09	0.07	0.11	0.09	0.08
		−0.01, 0.37	−0.08, 0.26	−0.09, 0.24	−0.06, 0.28	−0.08, 0.25	−0.08, 0.25
	>5–10 years	−0.08	−0.12	−0.16	−0.10	−0.13	−0.15
		−0.31, 0.15	−0.35, 0.10	−0.38, 0.06	−0.32, 0.12	−0.36, 0.09	−0.37, 0.06
	>10–15 years	−0.06	−0.10	−0.09	−0.08	−0.08	−0.06
		−0.37, 0.26	−0.40, 0.21	−0.38, 0.19	−0.38, 0.21	−0.39, 0.22	−0.35, 0.21
	>15 years	−0.72∗	−0.50∗	−0.15	−0.47∗	−0.47∗	−0.12
		−1.18, −0.26	−0.93, −0.07	−0.62, 0.31	−0.90, −0.05	−0.89, −0.05	−0.58, 0.34
**Processing speed (n = 875)**	1–5 years	0.10	0.06	0.04	0.08	0.07	0.08
		−0.11, 0.30	−0.14, 0.26	−0.16, 0.25	−0.12, 0.27	−0.13, 0.27	−0.13, 0.28
	>5–10 years	0.17	0.14	0.14	0.15	0.15	0.16
		−0.09, 0.4	−0.11, 0.39	−0.12, 0.39	−0.10, 0.40	−0.10, 0.39	−0.09, 0.42
	>10–15 years	−0.17	−0.17	−0.18	−0.17	−0.13	−0.13
		−0.45, 0.12	−0.44, 0.11	−0.46, 0.10	−0.44, 0.10	−0.41, 0.15	−0.40, 0.15
	>15 years	−0.61∗	−0.56∗	−0.58∗	−0.55∗	−0.49∗	−0.51∗
		−1.00, −0.23	−0.96, −0.15	−1.10, −0.06	−0.95, −0.15	−0.88, −0.09	−1.02, −0.004
**WOMEN**							

**Verbal memory (n = 934)**	1–5 years	−0.16	−0.13	−0.09	−0.11	−0.17	−0.13
		−0.40, 0.10	−0.35, 0.09	−0.31, 0.13	−0.33, 0.10	−0.38, 0.04	−0.34, 0.09
	>5–10 years	0.08	0.03	0.06	0.04	0.003	0.03
		−0.18, 0.33	−0.18, 0.25	−0.15, 0.28	−0.17, 0.26	−0.20, 0.21	−0.18, 0.24
	>10–15 years	−0.13	−0.13	−0.09	−0.10	−0.14	−0.09
		−0.41, 0.15	−0.36, 0.11	−0.33, 0.16	−0.33, 0.14	−0.37, 0.08	−0.33, 0.14
	>15 years	−0.22	−0.09	−0.04	−0.08	−0.12	−0.08
		−0.547, 0.03	−0.31, 0.12	−0.27, 0.18	−0.30, 0.14	−0.33, 0.09	−0.30, 0.13
**Processing speed (n = 949)**	1–5 years	−0.07	−0.09	−0.08	−0.07	0.07	−0.06
		−0.37, 0.22	−0.38, 0.20	−0.37, 0.20	−0.36, 0.21	−0.13, 0.27	−0.35, 0.22
	>5–10 years	−0.09	−0.14	−0.11	−0.13	0.15	−0.10
		−0.37, 0.19	−0.42, 0.13	−0.38, 0.16	−0.40, 0.14	−0.10, 0.39	−0.37, 0.17
	>10–15 years	−0.11	−0.12	−0.08	−0.08	−0.13	−0.04
		−0.43, 0.21	−0.44, 0.19	−0.40, 0.23	−0.39, 0.23	−0.41, 0.15	−0.36, 0.28
	>15–20 years	−0.13	−0.11	−0.05	−0.09	−0.49∗	−0.04
		−0.42, 0.17	−0.40, 0.19	−0.36, 0.25	−0.38, 0.20	−0.88, −0.09	−0.35, 0.27

Model 2: adjusted for childhood factors: childhood social class, cognitive ability (age 8), adolescent mental health, and educational attainment.

Model 3: model 2 additionally adjusted for the indicators of disuse: head of household social class and occupational complexity.

Model 4: model 2 additionally adjusted for the physical and mental health covariates (hypertension, BMI, mental health).

Model 5: model 2 additionally adjusted for the lifestyle covariates (smoking intensity, physical activity and social activity).

Final model: adjusted for the covariates above.

∗p < .05.

In women, none of the non-employment categories were associated with cognitive function at age 60–64.

### 3.3 Non-employment duration and cognitive decline

For men, while there was no association in models 1 and 2, the final models indicated that, when compared to 0–12 months non-employment, >10–15 years non-employment was associated with faster verbal memory decline (−0.34 s d. units, 95%CI: −0.75, −0.22 in the final model). The intermediary models suggested that the association was strengthened following adjustment for the indicators of disuse (model 3) and lifestyle (model 5). The unadjusted model also indicated that >5–10 years non-employment was associated with faster processing speed decline (−0.31 s d. units, 95%CI: −0.54, −0.07). In contrast to the association between >10–15 years non-employment and faster memory decline, this association was partially attenuated following adjustment for the indicators of disuse (model 3) and lifestyle (model 5), but the association remained in the final model (−0.28 s d. units, 95%CI: −0.52, −0.03) (Table 4).

**Table 4:** The association between non-employment and cognitive decline (standardised).

Non-employment duration *(reference category 0–*12 months *omitted)*		Model 1	Model 2	Model 3	Model 4	Model 5	Final model
		β	β	β	β	β	β
		95%CI	95%CI	95%CI	95%CI	95%CI	95%CI
**MEN**							

**Verbal recall (n = 854)**	1–5 years	−0.13	−0.13	−0.14	−0.12	−0.13	−0.13
		−0.33, 0.08	−0.33, 0.07	−0.34, 0.06	−0.32, 0.29	−0.32, 0.07	−0.32, 0.07
	>5–10 years	0.06	0.05	0.05	0.05	0.07	0.08
		−0.17, 0.29	−0.19, 0.29	−0.19, 0.29	−0.19, 0.29	−0.16, 0.30	−0.16, 0.32
	>10–15 years	−0.25	−0.27	−0.34	−0.28	−0.30	−0.38∗
		−0.50, 0.08	−0.62, 0.07	−0.69, 0.01	−0.65, 0.09	−0.65, 0.04	−0.75, −0.02
	>15 years	0.24	0.22	−0.03	0.20	0.22	−0.07
		−0.21, 0.69	−0.24, 0.68	−0.62, 0.57	−0.27, 0.66	−0.25, 0.68	−0.68, 0.55
**Search speed (n = 875)**	1–5 years	−0.13	−0.12	−0.12	−0.11	−0.12	−0.12
		−0.33, 0.07	−0.32, 0.09	−0.33, 0.08	−0.31, 0.09	−0.32, 0.08	−0.32, 0.08
	>5–10 years	−0.31∗	−0.29∗	−0.28∗	−0.29∗	−0.28∗	−0.28∗
		−0.54, −0.07	−0.52, −0.06	−0.51, −0.04	−0.52, −0.06	−0.51, −0.05	−0.52, −0.03
	>10–15 years	0.03	0.03	0.01	0.03	0.01	−0.005
		−0.26, 0.32	−0.27, 0.32	−0.30, 0.32	−0.27, 0.32	−0.28, 0.31	−0.32, 0.31
	>15 years	0.08	0.07	0.13	0.08	0.09	0.14
		−0.31, 0.47	−0.33, 0.47	−0.36, 0.62	−0.31, 0.47	−0.31, 0.48	−0.34, 0.63
**WOMEN**							

**Verbal recall (n = 934)**	1–5 years	0.21	0.22	0.22	0.23	0.23	0.24
		−0.04, 0.46	−0.03, 0.47	−0.04, 0.48	−0.02, 0.48	−0.02, 0.49	−0.02, 0.50
	>5–10 years	0.13	0.13	0.15	0.14	0.15	0.17
		−0.13, 0.38	−0.12, 0.38	−0.10, 0.40	−0.11, 0.39	−0.10, 0.41	−0.08, 0.43
	>10–15 years	0.22	0.22	0.22	0.23	0.24	0.24
		−0.06. 0.50	−0.06, 0.51	−0.07, 0.51	−0.06, 0.51	−0.05, 0.53	−0.05, 0.53
	>15 years	0.25	0.25	0.24	0.26	0.27	0.28
		−0.01, 0.52	−0.02, 0.51	−0.04, 0.52	−0.003, 0.53	−0.001, 0.54	−0.002, 0.56
**Search speed (n = 949)**	1–5 years	−0.15	−0.15	−0.17	−0.14	−0.18	−0.18
		−0.44, 0.14	−0.44, 0.14	−0.46, 0.12	−0.44, 0.15	−0.48, 0.12	−0.49, 0.12
	>5–10 years	−0.03	−0.01	−0.03	−0.0007	−0.04	−0.04
		−0.31, 0.25	−0.30, 0.27	−0.31, 0.25	−0.28, 0.28	−0.32, 0.25	−0.33, 0.24
	>10–15 years	0.20	0.19	0.17	0.2	0.18	0.16
		−0.12, 0.52	−0.12, 0.51	−0.15, 0.48	−0.11, 0.51	−0.14, 0.49	−0.16, 0.47
	>15 years	−0.09	−0.10	−0.12	−0.08	−0.12	−0.13
		−0.36, 0.17	−0.37, 0.18	−0.41, 0.16	−0.36, 0.19	−0.40, 0.15	−0.42, 0.16

Model 2: adjusted for childhood factors: childhood social class, cognitive ability (age 8), adolescent mental health, and educational attainment.

Model 3: model 2 additionally adjusted for the indicators of disuse: head of household social class and occupational complexity.

Model 4: model 2 additionally adjusted for the physical and mental health covariates (hypertension, BMI, mental health).

Model 5: model 2 additionally adjusted for the lifestyle covariates (smoking intensity, physical activity and social activity).

Final model: adjusted for the covariates above.

∗p < .05.

Among women, there was little evidence that non-employment was associated with cognitive decline; if anything, increasing non-employment duration was associated with slower cognitive decline in later life.

## 4 Discussion

This study examined the longitudinal associations of non-employment with cognitive function and decline in later life among men and women in Britain, using data from a birth cohort study. It examined these associations separately in men and women.

The finding that long-term non-employment was associated with lower cognitive function among men, and that this association was partially explained by the indicators of disuse was consistent with our first hypothesis. It reflected the findings of studies that have found that engaging in everyday cognitive activities was associated with higher cognitive function in later life [7,[32], [33], [34]]; and studies showing that high complexity occupations were associated with higher cognitive function taking into account a range of socio-economic and health factors [15,35,36]. Furthermore, it corroborated Adam et al., 2013 which showed that study members who experienced longer non-employment durations prior to retirement age scored lower on cognitive tests than study members who continued to work up to retirement age, and this difference was compensated for when specific non-professional activities were included in the model [18].

Confounding by common cause from childhood factors and educational attainment explained some of the association in men, suggesting a pathway from a disadvantaged childhood and low educational attainment to disadvantaged social circumstances and increased risk of non-employment, and in parallel to lower cognitive function. However, residual negative associations remained between long-term non-employment and lower cognitive function, which may reflect additional factors that we could not test, including loss of daily structure, identity, and interaction [37].

Our finding that long-term non-employment was associated with faster cognitive decline in men but not women also partially supported our first hypothesis. It partially replicated those studies reporting that high complexity occupations were associated with slower cognitive decline [16,38]. However, it contrasted with Leist et al.’s (2013) finding that being unemployed and seeking work was not associated with faster cognitive decline [17]. However, their study focused on being unemployed and looking for a job, rather than non-employment in general, and measured decline over a two-year period, which may have been too short for any meaningful change in cognitive function to be observed.

We also confirmed our second hypothesis that associations between non-employment and cognitive function and decline would be stronger in men than women. These gender differences may be understood in terms of the gendered division of labour affecting this cohort [39], with the emphasis being on women as caregivers, responsible for domestic work. For the majority of women in this cohort, work was a temporary state prior to exit from employment to marry and raise a family. While many did return to the workforce, their jobs tended to be part-time and of lower value than that of men [19]. Long durations of non-employment for women were therefore common and women may also have benefited from alternative sources of cognitive engagement e.g. from childrearing, including school-oriented activities. In contrast, male non-employment of ≥15 years was rare, with the majority working for approximately 50 years. Furthermore, those men who were non-employed for <15 years experienced most of their non-employment after age 50. Therefore, longer non-employment durations could have represented retirement prior to age 50, which would have been non-normative for men in this cohort.

### 4.1 Limitations

Our study had several limitations. Employment information was reported retrospectively, although this was limited to an interval of ≤15 years, decreasing likelihood of recall error. Retest (practice) effects on the cognitive tests may have resulted in cognitive decline being underestimated. However, studies have indicated that retest effects are limited beyond the first two test occasions [40], and our analyses did not use the cognitive data from the first two data collection waves at age 43 and 53. Furthermore, two word lists were used for verbal memory and were alternated between waves, addressing the possibility that study members would remember those that were presented at the previous wave.

Additionally, we were not able to account for different types of non-employment, which may differ in the cognitive stimulation that they provide. For example, education/training may provide more cognitive stimulation than sickness or disability. Although this information on the type of non-employment was collected during at ages 26, 36 and 43, it was not collected during the later data collections, and therefore, these differences could not be examined. Future research using longitudinal studies of ageing could investigate this and could therefore investigate whether the association between long-term non-employment and lower cognitive function was associated with sickness and disability non-employment. However, these longitudinal studies of ageing tend to have greater reliance on retrospectively collected non-employment information and are not able to adjust for early life cognitive ability.

A final limitation was that these findings may not be generalisable to later generations of women owing to the changing nature of their work. Women's labour force participation is increasing, which may translate into an increasing importance of employment in shaping women's cognitive abilities and functioning in later life. Therefore the associations documented here may change among more recently born generations of women. Further research should investigate this using data from the more recent British birth cohorts (the National Child Development Study (NCDS), 1958 Birth Cohort; and the British Cohort Study (BCS), 1970 Birth Cohort), examining whether the association between non-employment and cognitive function and decline in women is changing.

### 4.2 Strengths

Nonetheless, our study has several strengths, above all the use of a nationally representative population-based study with repeated measures of various cognitive tests, long periods of follow-up, and detailed information on non-employment and a wide range of important prospectively collected covariates, including the rarely-available childhood cognition. The cognitive outcomes assessed two core aspects of fluid ability that are sensitive to age, episodic memory and psychomotor speed. Use of multiple imputation analysis was a further strength, reducing bias attributable to missing information. A final strength was that the analyses were gender specific. Previous studies have adjusted for sex, not allowing for the possibility that the different employment experiences of men and women may translate into gender differences in the associations between non-employment and cognition.

## 5 Conclusion

In summary, this research indicated that among men, long-term non-employment over the working life was associated with lower cognitive function and faster cognitive decline in later life. Policies that provide meaningful cognitive engagement for non-employed men both during their working life and post-retirement need to be developed. Meaningful activities such as education and training, book clubs and similarly engaging lifestyle activities during non-employment spells may provide alternative means of cognitive engagement and new goal structures, decreasing non-employment's impact on cognitive function and possibly decline. Although non-employment among women was not associated with cognitive function or decline in this cohort, this may be different for more recently born cohorts of women, who are more engaged with the labour market. Therefore, similar policies may need to be considered for women who are currently working age.

## Ethical approval

Ethical approval for the last two data collections in 2006-10 and 2014-16 was obtained from the Multicentre Research Ethics Committee, and from Queens Square Research Ethics Committee (14/LO/1073) and the Scotland A Research Ethics Committee (14/SS/1009). All study members gave written informed consent.

## Funding

AJS was funded by the UK 10.13039/501100000269Economic and Social Research Council (grant no. ES/J500185/1).

AS was funded by the UK 10.13039/501100000269Economic and Social Research Council (grant no. ES/J019119/1).

REL was funded by the UK 10.13039/501100000269Economic and Social Research Council (grant no. ES/P010229/1).

MR was funded by the 10.13039/501100000265UK Medical Research Council (Unit Programme number MC UU 12019/8).

## Conflict of interest

No conflicts of interest to report.

## Footnotes

1 SBP, FEV1 and self-reported heart problems did not improve the fit of model 1 and were not included in model 4.
