# Supplementary material for: Non-employment over the working life: Implications for cognitive function and decline in later life
Source: Public Health Pract (Oxf). 2024 Dec 23;9:100563. doi: 10.1016/j.puhip.2024.100563 (PMC11758421; doi:10.1016/j.puhip.2024.100563)
Supplement: Multimedia component 1 [file mmc1.docx]

**Supplementary table 1: Imputed versus observed values of the covariates**

| **Variable** | **Men** | | **Women** | |
| --- | --- | --- | --- | --- |
|  | **Observed** | **Imputed** | **Observed** | **Imputed** |
| **Cognitive function** |  |  |  |  |
| Verbal recall (age 60+) | 22.68 | 22.68 | 24.92 | 24.92 |
|  | 22.23, 23.14 | 22.23, 23.14 | 24.48, 25.37 | 24.47, 25.37 |
| Verbal recall (age 69) | 20.78 | 20.78 | 22.84 | 22.84 |
|  | 20.32, 21.24 | 20.32, 21.24 | 22.40, 23.28 | 22.40, 23.28 |
| Search speed (age 60+) | 259.51 | 259.51 | 267.83 | 267.83 |
|  | 253.99, 265.04 | 253.99, 265.04 | 262.17, 273.50 | 262.17, 273.50 |
| Search speed (age 69) | 252.20 | 252.20 | 263.86 | 263.86 |
|  | 246.69, 257.71 | 246.69, 257.71 | 258.25, 269.47 | 258.25, 269.47 |
| **Non-employment duration** |  |  |  |  |
| 0-12 months | 53.14 | 50.47 | 17.88 | 15.94 |
|  | 48.62, 57.60 | 46.23, 54.71 | 14.81, 21.44 | 13.04, 18.83 |
| >1-5 years | 23.03 | 23.29 | 27.03 | 24.81 |
|  | 19.48, 27.01 | 19.71, 26.88 | 23.40, 31.00 | 21.40, 28.22 |
| >5-10 years | 14.62 | 15.77 | 22.91 | 22.67 |
|  | 11.72, 18.09 | 12.64, 18.89 | 19.54, 26.66 | 19.23, 26.12 |
| >10-15 years | 6.21 | 6.84 | 14.17 | 16.20 |
|  | 4.35, 8.79 | 4.61, 9.07 | 11.46, 17.41 | 13.05, 19.34 |
| >15-20 years | 3.00 | 3.63 | 9.69 | 10.37 |
|  | 1.76, 5.08 | 1.99, 5.26 | 7.34, 12.68 | 7.75, 13.00 |
| >20 years |  |  | 8.31 | 10.01 |
|  |  |  | 6.22, 11.03 | 7.56, 12.45 |
| **Childhood social class** |  |  |  |  |
| I: Professional and managerial | 5.17 | 5.09 | 3.62 | 3.63 |
|  | 3.97, 6.70 | 3.75, 6.44 | 2.76, 4.73 | 2.61, 4.65 |
| II: Intermediate | 16.68 | 16.60 | 17.34 | 17.33 |
|  | 14.02, 19.72 | 13.77, 19.43 | 14.74, 20.28 | 14.56, 20.10 |
| IIINM: Skilled non-manual | 10.39 | 10.36 | 11.31 | 11.29 |
|  | 8.57, 12.54 | 8.36, 12.36 | 9.36, 13.60 | 9.15, 13.44 |
| IIIM: Skilled manual | 43.12 | 43.28 | 44.62 | 44.63 |
|  | 38.99, 47.35 | 39.10, 47.46 | 40.67, 48.65 | 40.66, 48.09 |
| IV: Semi-skilled | 16.62 | 16.59 | 17.84 | 17.75 |
|  | 13.70, 20.02 | 13.45, 19.94 | 15.00, 21.09 | 14.74, 20.76 |
| V: Unskilled | 8.03 | 8.08 | 5.28 | 5.36 |
|  | 5.90, 10.83 | 5.65, 10.50 | 3.67, 7.53 | 3.45, 7.27 |
| **Childhood cognitive ability** | -0.002 | 0.005 | 0.05 | 0.06 |
|  | -0.07, 0.07 | -0.006, 0.07 | -0.01, 0.11 | -0.003, 0.12 |
| **Adolescent mental health** |  |  |  |  |
| Self-organisation | 1.00 | 0.98 | 1.41 | 1.40 |
|  | 0.88, 1.11 | 0.86, 1.09 | 1.30, 1.52 | 1.30, 1.51 |
| Emotional problems | -0.24 | -0.22 | 0.04 | 0.02 |
|  | -0.35, -0.12 | -0.34, -0.11 | -0.07, 0.15 | -0.09, 0.13 |
| Control problems | 0.18 | 0.20 | -0.09 | -0.07 |
|  | 0.07, 0.30 | 0.09, 0.32 | -0.22, 0.03 | -0.19, 0.05 |
| **Educational attainment** |  |  |  |  |
| None attempted | 35.20 | 34.92 | 34.76 | 35.34 |
|  | 31.25, 39.37 | 30.90, 38.94 | 31.03, 38.69 | 31.53, 39.15 |
| Sub GCE or sub Burnham | 5.56 | 5.61 | 11.95 | 11.85 |
|  | 3.90, 7.86 | 3.65, 7.57 | 9.52, 14.90 | 9.20, 14.50 |
| GCE O’ Level and equivalent | 14.97 | 14.99 | 26.25 | 26.03 |
|  | 12.26, 18.15 | 12.05, 17.92 | 22.94, 29.84 | 22.61, 29.45 |
| GCE A’ Level and equivalent | 30.73 | 31.00 | 22.56 | 22.35 |
|  | 27.05, 34.67 | 27.18, 34.81 | 19.62, 25.80 | 19.30, 25.41 |
| 1st degree, graduate equivalent and above | 13.55 | 13.48 | 4.48 | 4.43 |
|  | 11.20, 16.30 | 10.94, 16.02 | 3.25, 6.16 | 3.01, 5.84 |
| **Head of household social class** |  |  |  |  |
| I: Professional (prof.) and managerial | 7.35 | 7.28 | 6.50 | 6.42 |
|  | 5.57, 9.64 | 5.29, 9.27 | 4.82, 8.71 | 4.52, 8.32 |
| II: Intermediate | 25.83 | 25.70 | 23.03 | 22.97 |
|  | 22.39, 29.60 | 22.18, 29.23 | 19.89, 26.50 | 19.66, 26.28 |
| IIINM: Skilled non-manual | 8.05 | 7.92 | 11.16 | 11.21 |
|  | 6.03, 10.66 | 5.67, 10.18 | 8.92, 13.87 | 8.74, 13.68 |
| IIIM: Skilled manual | 23.09 | 22.96 | 9.83 | 9.83 |
|  | 19.67, 26.91 | 19.43, 26.48 | 7.64, 12.56 | 7.39, 12.26 |
| IV: Semi-skilled | 6.47 | 6.45 | 5.68 | 5.57 |
|  | 4.59, 9.06 | 4.25, 8.64 | 4.02, 7.97 | 3.67, 7.47 |
| V: Unskilled | 2.39 | 2.45 | 1.02 | 1.01 |
|  | 1.37, 4.14 | 1.11, 3.80 | 0.46, 2.28 | 0.19, 1.83 |
| Not working | 26.82 | 27.23 | 42.78 | 43.00 |
|  | 23.23, 30.75 | 23.47, 31.00 | 38.90, 46.76 | 39.04, 46.95 |
| **NS-SEC (occupational complexity)** |  |  |  |  |
| Higher managerial. administrative, and prof. | 19.50 | 19.50 | 3.07 | 3.17 |
|  | 16.61, 22.75 | 16.44, 22.57 | 2.05, 4.57 | 1.89, 4.44 |
| Lower managerial. administrative, and prof. | 22.16 | 22.20 | 27.30 | 27.29 |
|  | 19.05, 25.62 | 18.92, 25.48 | 24.07, 30.78 | 23.93, 20.64 |
| Intermediate | 6.41 | 6.42 | 24.72 | 24.61 |
|  | 4.71, 8.67 | 4.46, 8.37 | 21.51, 28.24 | 21.25, 27.98 |
| Small employers and own account workers | 19.88 | 19.81 | 10.56 | 10.55 |
|  | 16.79, 23.39 | 16.52, 23.09 | 8.39, 13.22 | 8.15, 12.94 |
| Lower supervisory and technical workers | 9.78 | 9.79 | 3.07 | 3.09 |
|  | 7.51, 12/63 | 7.25, 12.33 | 1.94, 4.81 | 1.69, 4.48 |
| Semi-routine | 11.14 | 11.10 | 21.46 | 21.42 |
|  | 8.71, 14.14 | 8.41, 13.79 | 18.39, 24.89 | 18.17, 24.67 |
| Routine | 11.14 | 11.18 | 9.83 | 9.88 |
|  | 8.74, 14.09 | 8.51, 13.85 | 7.69, 12.48 | 7.49, 12.27 |
| **Heart problems** |  |  |  |  |
| No | 75.44 | 74.95 | 75.05 | 74.20 |
|  | 71.54, 78.96 | 71.27, 78.63 | 71.41, 78.37 | 70.75, 77.66 |
| Yes | 24.56 | 25.05 | 24.95 | 25.80 |
|  | 21.04, 28.46 | 21.37, 28.73 | 21.63, 28.59 | 22.34, 29.25 |
| **Hypertension** |  |  |  |  |
| No | 63.73 | 63.66 | 64.03 | 63.38 |
|  | 59.56, 67.70 | 59.63, 67.69 | 60.11, 67.78 | 59.56, 67.21 |
| Yes | 36.27 | 36.34 | 35.97 | 36.62 |
|  | 32.30, 40.44 | 32.31, 40.37 | 32.22, 39.89 | 32.79, 40.44 |
| **SBP (adjusted)** | 143.35 | 143.35 | 135.57 | 135.58 |
|  | 141.77, 144.93 | 141.77, 144.93 | 134.04, 137.05 | 134.10, 137.06 |
| **FEV_1_** | 3.07 | 3.05 | 2.13 | 2.12 |
|  | 3.01, 3.12 | 3.00, 3.11 | 2.10, 2.17 | 2.09, 2.16 |
| **BMI** | 27.92 | 27.92 | 18.39 | 28.45 |
|  | 27.61, 28.24 | 27.61, 28.24 | 27.95, 28.84 | 27.99, 28.91 |
| **Mental health** | 1.74 | 1.74 | 2.86 | 2.85 |
|  | 1.49, 2.00 | 1.49, 2.00 | 2.51, 3.21 | 2.50, 3.20 |
| **Smoking (pack years)** | 13.01 | 12.84 | 9.85 | 9.73 |
|  | 11.46, 14.56 | 11.30, 14.37 | 8.53, 11.17 | 8.45, 11.00 |
| **Physical activity** |  |  |  |  |
| 0: Inactive (all ages) | 23.33 | 23.91 | 28.63 | 28.73 |
|  | 20.02, 27.00 | 20.31, 27.52 | 25.23, 32.28 | 25.14, 32.32 |
| 1: Inactive/ low activity (all ages) | 27.42 | 27.56 | 31.07 | 31.31 |
|  | 23.94, 31.21 | 23.70, 31.43 | 27.60, 34.76 | 27.62, 35.00 |
| 2: Low - moderate activity | 26.19 | 24.70 | 21.01 | 20.58 |
|  | 22.82, 29.86 | 21.11, 28.29 | 18.11, 24.25 | 17.43, 23.73 |
| 3: Moderate activity | 15.73 | 17.14 | 14.03 | 14.70 |
|  | 13.01, 18.90 | 14.03, 20.25 | 11.61, 16.85 | 11.92, 17.48 |
| 4: High activity (all ages) | 7.33 | 6.68 | 5.27 | 4.67 |
|  | 5.57, 9.58 | 4.71, 8.66 | 3.79, 7.27 | 3.02, 6.33 |
| **Social activity** |  |  |  |  |
| 0 activities/ month | 24.33 | 24.58 | 23.02 | 23.49 |
|  | 20.81, 28.23 | 20.86, 28.29 | 19.75, 26.64 | 20.35, 26.94 |
| 1 activity/ month | 27.3 | 27.33 | 24.04 | 24.16 |
|  | 23.65, 31.27 | 23.53, 31.31 | 20.76, 27.66 | 20.71, 27.61 |
| 2 activities/ month | 23.92 | 23.82 | 22.4 | 22.21 |
|  | 20.55, 27.66 | 20.22, 27.42 | 19.29, 25.87 | 18.93, 25.50 |
| 3 activities/ month | 24.45 | 24.28 | 30.54 | 30.14 |
|  | 21.39, 28.09 | 20.82, 27.72 | 27.08, 34.23 | 26.60, 33.69 |
